# Comparison of Colour Duplex Ultrasound with Computed Tomography to Measure the Maximum Abdominal Aortic Aneurysmal Diameter

**DOI:** 10.1155/2014/574762

**Published:** 2014-11-23

**Authors:** C. Gray, P. Goodman, S. A. Badger, M. K. O'Malley, M. K. O'Donohoe, C. O. McDonnell

**Affiliations:** ^1^Department of Vascular Surgery, Mater Misericordiae University Hospital, Dublin, Ireland; ^2^Dublin School of Physics, Dublin Institute of Technology, Dublin, Ireland

## Abstract

*Introduction*. Maximum diameter of an abdominal aortic aneurysm (AAA) is the main indication for surgery. This study compared colour duplex ultrasound (CDU) and computed tomography (CT) in assessing AAA diameter. *Patients and Methods*. Patients were included if they had both scans performed within 90 days. Pearson's correlation coefficient, paired *t*-test, and limits of agreement (LOA) were calculated for the whole group. Subgroup analysis of small (<5.0 cm), medium (5.0–6.5 cm), and large (>6.5 cm) aneurysms was performed. A *P* value of <0.05 was considered statistically significant. *Results*. 389 patients were included, giving 130 pairs of tests for comparison. Excellent correlation was in the whole group (*r* = 0.95) and in the subgroups (*r* = 0.94; 0.69; 0.96, resp.). Small LOA between the two imaging modalities was found in all subgroups. *Conclusion*. Small aneurysms can be accurately measured using CDU. CDU is preferable for small AAAs, but cannot supplant CT for planning aortic intervention.

## 1. Introduction

Annual abdominal aortic aneurysm (AAA) maximum aneurysm diameter is the main indication for the timing of elective repair. The modality of choice to determine the maximum diameter prior to intervention is computed tomography (CT). CT provides a detailed anatomical image with 3-dimensional reconstruction, thus allowing measurement of the maximal aortic diameter perpendicular to the central line of flow [1]. CT is considered to be more reproducible than CDU, with more than 90% of remeasurements being within 2 mm of the initial figure [2]. Intervention often is triggered by a maximum diameter of greater than 5.5 cm [3–5]. Despite its advantages, CT is both expensive and associated with certain risks to the patient in terms of radiation exposure and intravenous contrast administration. The need for multiple scans for AAA surveillance increases this risk. The majority of intravenous contrast agents currently in use are iodine based and adverse reactions such as contrast nephropathy or anaphylaxis.

Colour duplex ultrasound (CDU) is a safe modality for the surveillance of patients with small AAA's [5, 6]. It is noninvasive with a sensitivity of 95% and a specificity approaching 100% when performed in a setting with adequate quality assurance [7]. CDU is less expensive, more widely available and has no exposure to radiation or intravenous contrast. The investigation can also be conducted as a portable examination, allowing the scanner to travel to the patient, rather than the patient travel to the scanner, if necessary.

This study aimed to compare the two imaging modalities of CDU and CT in assessment of the maximum aneurysm diameter in patients under surveillance for AAA.

## 2. Patients and Methods

Approval was obtained from the hospital ethics committee for the study. All patients attending the vascular laboratory of the Mater Misericordiae University Hospital (MMUH) between the 1st of January 2007 and the 31st of December 2009 were recruited if they had a CDU and a CT scan for assessment of their AAA within 90 days of each other.

### 2.1. Colour Duplex Ultrasound Imaging

All CDU scans were performed in the supine position by a qualified vascular technologist proficient in abdominal imaging using one of 3 machines, a Siemens Sequoia 512, a Siemens S2000, or a Phillips IU22. All CDU scans were performed using a wideband curvilinear transducer. The maximum anterior to posterior (AP) wall diameter and the maximum transverse wall diameter were recorded with the greater of the two measurements being taken as the maximum aneurysm diameter and used for comparison in this study. The outer-to-outer diameter was used for the definition of AAA diameter.

### 2.2. Computed Tomography

All CT scans were carried out in the Radiology Department of the MMUH following their standard protocol for abdominal imaging. The maximum aneurysm diameter documented on the final report by a consultant radiologist was used for comparison in this study. The outer-to-outer diameter was also used as the diameter definition for CT scans, to ensure equality of definition in comparison.

### 2.3. Statistical Analysis

Continuous variables were expressed as mean (± SD). Correlation between the CDU and CT was performed using Pearson's coefficient correlation analysis. Limits of agreement (LOA) were also performed with the method described by Bland and Altman [8]. LOA comprises two values, a positive (LOA-P) and a negative (LOA-N), that define the range in which 95% of the differences between the methods of measurements fall [9]. In this study, the LOA was calculated using MedCalc statistical software and was calculated as the mean difference ± 1.96 times, the standard deviation of the differences. The accepted value for LOA is between −0.5 and 0.5 cm, which are the values between which 95% of the measured differences are expected to fall.

**Figure 1:**
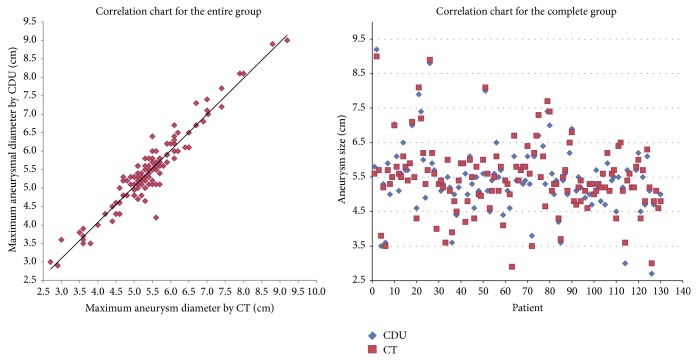
Correlation chart for the complete cohort showing a large degree of correlation between CDU and CT.

Pearson's correlation coefficient, paired* t*-test, and LOA were calculated for the group of patients as a whole. Patients were then divided into three subgroups small, medium, and large aneurysms (Figure 1) [9]. A *P* value of less than 0.05 was considered significant.

## 3. Results

During the study period, 389 patients attended the vascular laboratory for aortic aneurysm surveillance. Of these, 126 had both scans performed within 90 days of each other. The remaining 263 patients were excluded as they did not have comparable scans within the 90-day period. In all cases, this was because these aneurysms fell below the standard threshold for intervention of 5.5 cm and thus a CT was not warranted. Due to multiple scans within the study period in 4 patients, a total of 130 pairs of tests are available for comparison. Ninety-nine patients (78.6%) were male and twenty-seven (21.4%) were female with an overall mean age of 76.1 (± 7.1) years. The mean male age was 76.1 (± 6.5) and the mean female age was 76.2 (± 9.0).

### 3.1. Correlation between Modalities of Measurements

*Entire Group (n* = 130). Mean AAA diameter on CDU was 5.4 (± 1.0) cm and on CT was 5.4 (± 1.0) cm. Correlation was excellent (*r* = 0.95; Figure 1). There was no statistical difference between the two modalities in diameter measurement (*P* = 0.10).

*Small Aneurysms (A* *AA* < 5.0* cm)*. Twenty-nine pairs of scans were in this subgroup. Mean AAA diameter on CDU was 4.2 (± 0.68) cm and 4.2 (± 0.58) cm on CT (*P* = 0.4). Correlation was excellent (*r* = 0.94) for aneurysms less than 5.0 cm.

*Medium Aneurysms (AAA *5.0*>*–*<*6.5* cm)*. Eighty-eight (69.8%) pairs of scans were in this subgroup. Mean aneurysm size on CDU was 5.5 (±0.39) cm and was 5.0 (± 0.43) cm on CT (*P* = 0.2). Correlation for this group was good (*r* = 0.69).

*Large Aneurysms (*>6.5* cm)*. Thirteen pairs of scans were in this subgroup. Mean AAA diameter on CDU was 7.4 (± 0.83) cm and 7.5 (± 0.79) cm on CT (*P* = 0.1). Correlation was found excellent (*r* = 0.96).

## 4. Limits of Agreement

### 4.1. Overall Cohort

**Figure 2:**
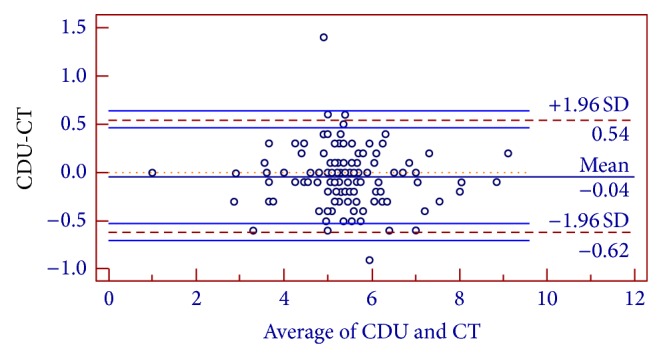
Bland and Altman plot for the whole cohort.

Figure 2 is the mean differences between the two measurements plotted against the mean aneurysmal diameter. The limits of agreement were found to be −0.62–0.54, indicating a 95% confidence level that the error between the two techniques is within this range. However, the LOA is outside the accepted range between −0.5 and 0.5.

### 4.2. Small Aneurysms

The limits of agreement were found to be between −0.46 and 0.47, within the acceptable range.

### 4.3. Medium Aneurysms

The limits of agreement were found to be between −0.68–0.59, within the acceptable range.

### 4.4. Large Aneurysms

The limits of agreement were found to be between −0.55–0.35, within the acceptable range.

## 5. Discussion

This study evaluated the accuracy of CDU in assessing maximum AAA diameter compared to the gold standard method of CT. The two modalities demonstrated a large overall degree of correlation and a strong correlation achieved in all three subgroups by size, verifying that measurement of aneurysm size can be accurately measured by either imaging modality. LOA analysis of both the overall cohort and the subgroups demonstrated that despite achieving excellent correlation and small LOA between the two imaging modalities in all groups, the small aneurysm groups (<5 cm) were found to have better agreement with 95% of the differences in the two measurements falling between −0.46 and 0.47.

Discordance in measurements between various imaging modalities when measuring the maximal AAA diameter has been reported previously. Several authors have reported that maximal AAA diameter on CT is smaller than that obtained on duplex, while Meier documented that AAA diameter with ultrasound is usually larger than CT [9–11]. In a study by Manning the mean CT measurement was significantly larger than that of ultrasound with others reporting that ultrasound measurements are consistently smaller than those found on CT [4, 9, 11]. Measurement of maximal aneurysm diameter on CT is considered the most accurate method [12]. The reporting standards for endovascular aneurysm repair from the Society of Vascular Surgery recommended that AAA size be measured in three-dimensional reconstructions. However, amongst asymptomatic patients, ultrasound detects the presence of an aneurysm accurately, reproducibly and at a low cost with a sensitivity and specificity approaching 100%, with 1–3% of ultrasound scans being inconclusive due to the patient's body habitus or the presence of bowel gas. CT is more reproducible than ultrasound, but the advantages of ultrasound make it the method of choice for surveillance, with CT being the primary modality of choice for preoperative assessment.

The discordance between imaging modalities has been explained by the variation in techniques used to determine maximum aneurysm diameter, together with the presence of interobserver error. The definition of maximum diameter in this study for both modalities was outer-to-outer diameter. Inner-to-inner diameters were not measured in this study, and similar correlations are likely to be obtained, although it would be an interesting further study that could be performed. The United Kingdom Small Aneurysm Trial (UKSAT participants) used the maximal anterior to posterior wall measurements as obtained by ultrasound and recommended that surgical repair should take place on aneurysms greater than 5.5 cm in AP measurements [5, 6]. This study employed the method similar to that used in the Multi-Centre Aneurysm Screening Study (MASS), which measured both the maximal AP and transverse diameter, with the higher of the two measurements being reported as the AAA size [13].

The high degree of correlation achieved in this study may be explained by the improved greyscale resolution and harmonic imaging achieved by the currently available ultrasound machines. Duplex colour ultrasound was used in this study, as it is local protocol to do so, for the purposes of identifying any other haemodynamically significant coexisting occlusive diseases. However, B-mode sonography would provide equivalent measurements and correlations with CT, since it is the B-mode aspect of the scan that provides the measurements. Sprouse demonstrated an overall high correlation of 0.70 compared to the overall correlation of 0.95 found in this study [9]. They also found that despite obtaining a good degree of correlation, their LOA was clinically unacceptable at −0.45 to 2.36 cm compared to the −0.62–0.54 achieved in this study. Their subgroup analysis also demonstrated poor LOA in all cases.

## 6. Conclusion

AAA's less than 5 cm in diameter can be accurately measured using CDU. On the basis of these results, it is reasonable to suggest that CDU is the surveillance tool of choice for small AAAs, but cannot hope to supplant CT as the definitive planning tool prior to aortic intervention.
